# Micropeptide

**DOI:** 10.1371/journal.pgen.1007764

**Published:** 2018-12-13

**Authors:** Maria E. Sousa, Michael H. Farkas

**Affiliations:** 1 Ophthalmology, Jacobs School of Medicine and Biomedical Science, University of New York at Buffalo, Buffalo, NY, United States of America; 2 Research Service, Veterans Administration Western New York Healthcare System, Buffalo, NY, United States of America; 3 Department of Biochemistry, Jacobs School of Medicine and Biomedical Science, State University of New York at Buffalo, Buffalo, NY, United States of America

This is a Topic Page article for *PLOS Genetics*

## Introduction

**Fig 1:**
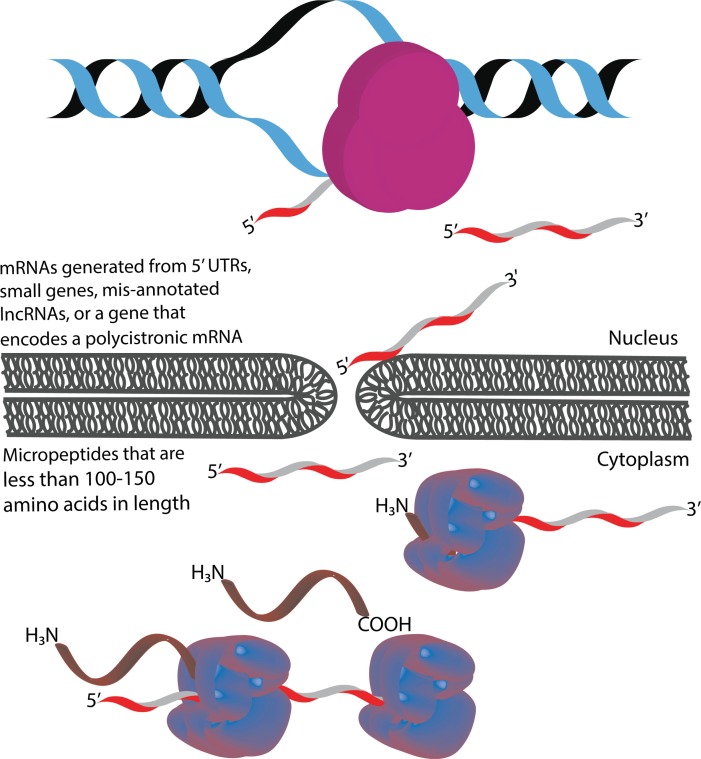
Genetic sources of micropeptides. Micropeptides can be transcribed from a small gene, a polycistronic mRNA, or from a lncRNA.

**Micropeptides** are commonly defined as polypeptides with a relatively arbitrary length of less than 100–150 amino acids (aa) in length [1–3]. They are distinguishable from bioactive peptides as the former is generated from short open reading frames (sORFs), whereas the latter is a cleavage product of a larger polypeptide [1,4].They differ from canonical proteins which have an average length of 330 and 449 amino acids in prokaryotes and eukaryotes, respectively[5]. Micropeptides, also known as microproteins or sORF-encoded peptides, can also be named according to their genomic location. For example, the translated product of an upstream open reading frame (uORF) might be called a uORF-encoded peptide (uPEP) [6].Micropeptides lack an N-terminal signaling sequence, suggesting they are likely to be localized to the cytoplasm after translation [1]. However, as more micropeptides are studied, they have been found in other cell compartments, as indicated by the existence of transmembrane micropeptides [7,8].They are expressed in both prokaryotic and eukaryotic organisms. [1,9,10]. The sORFs from which micropeptides are translated can be encoded in a variety of genomic regions. These include 5' UTRs, small genes, polycistronic mRNAs, or genes originally characterized as long non-coding RNAs (lncRNAs) (Fig 1) [11].

Given their small size, sORFs were originally overlooked. However, hundreds of thousands of putative micropeptides have been identified through various techniques in a multitude of organisms. Only a small fraction of these with coding potential have had their expression and function confirmed. Those that have been functionally characterized, in general, have roles in cell signaling, organogenesis, and cellular physiology. As more micropeptides are discovered so are more of their functions. One regulatory function is that of peptoswitches, which inhibit expression of downstream coding sequences by stalling ribosomes, through their direct or indirect activation by small molecules [11].

## Techniques for identifying potential micropeptides

Various experimental techniques exist for identifying sORFs and their translational products. It should be noted that these techniques are only useful for identification of sORF that may produce micropeptides and not for direct functional characterization.

### RNA-Sequencing

One method for finding potential sORFs, and therefore micropeptides, is through RNA-sequencing (RNA-Seq). RNA-Seq uses next-generation sequencing (NGS) to determine which RNAs are expressed in a given cell, tissue, or organism at a specific point in time. This collection of data, known as a transcriptome, can then be used as a resource for finding potential sORFs [1]. Because of the strong likelihood of sORFs less than 100 aa occurring by chance, further study is necessary to determine the validity of data obtained using this method [11].

### Ribosome profiling (Ribo-Seq)

Ribosome profiling has been used to identify potential micropeptides in a growing number of organisms, including fruit flies, zebrafish, mice and humans [11]. One method uses compounds such as harringtonine, puromycin or lactimidomycin to stop ribosomes at translation initiation sites [12]. This indicates where active translation is taking place. Translation elongation inhibitors, such as emetine or cycloheximide, may also be used to obtain ribosome footprints which are more likely to result in a translated ORF [13]. If a ribosome is bound at or near a sORF, it putatively encodes a micropeptide [1,2,14].

### Mass spectrometry

Mass Spectrometry (MS) is the gold standard for identifying and sequencing proteins. Using this technique, investigators are able to determine if polypeptides are, in fact, translated from a sORF.

### Proteogenomic applications

Proteogenomics combines proteomics, genomics, and transciptomics. This is important when looking for potential micropeptides. One method of using proteogenomics entails using RNA-Seq data to create a custom database of all possible polypeptides. Liquid chromatography followed by tandem MS (LC-MS/MS) is performed to provide sequence information for translation products. Comparison of the transcriptomic and proteomics data can be used to confirm the presence of micropeptides [1,2].

### Phylogenetic conservation

Phylogenetic conservation can be a useful tool, particularly when sifting through a large database of sORFs. The likelihood of a sORF resulting in a functional micropeptide is more likely if it is conserved across numerous species [11,12]. However, this will not work for all sORFs. For example, those that are encoded by lncRNAs are less likely to be conserved given lncRNAs themselves do not have high sequence conservation [2]. Further experimentation will be necessary to determine if a functional micropeptide is in fact produced.

## Validating protein-coding potential

### Antibodies

Custom antibodies targeted to the micropeptide of interest can be useful for quantifying expression or determining intracellular localization. As is the case with most proteins, low expression may make detection difficult. The small size of the micropeptide can also lead to difficulties in designing an epitope from which to target the antibody [2].

### Tagging with CRISPR-Cas9

Genome editing can be used to add FLAG/MYC or other small peptide tags to an endogenous sORF, thus creating fusion proteins. In most cases, this method is beneficial in that it can be performed more quickly than developing a custom antibody. It is also useful for micropeptides for which no epitope can be targeted [2].

### In vitro translation

This process entails cloning the full-length micropeptide cDNA into a plasmid containing a T7 or SP6 promoter. This method utilizes a cell-free protein-synthesizing system in the presence of ^35^S-methionine to produce the peptide of interest. The products can then be analyzed by gel electrophoresis and the ^35^S-labeled peptide is visualized using autoradiography [2].

## Databases and repositories

There are several repositories and databases that have been created for both sORFs and micropeptides.

A repository for of small ORFs discovered by ribosome profiling can be found at www.sorfs.org

A repository of putative sORF-encoded peptides in *Arabidopsis thaliana* can be found at ARA-PEPs

A database of small proteins, especially encoded by non-coding RNAs can be found at SmProt

## Prokaryotic examples

To date, most micropeptides have been identified in prokaryotic organisms. While most have yet to be fully characterized, of those that have been studied, many appear to be critical to the survival of these organisms. Because of their small size, prokaryotes are particularly susceptible to changes in their environment, and as such have developed methods to ensure their existence.

### *Escherichia coli* (*E. coli*)

Micropeptides expressed in *E*. *coli* exemplify bacterial environmental adaptations. Most of these have been classified into three groups: leader peptides, ribosomal proteins, and toxic proteins. Leader proteins regulate transcription and/or translation of proteins involved in amino acid metabolism when amino acids are scarce. Ribosomal proteins include L36 (*rpmJ*) and L34 (*rpmH*), two components of the 50S ribosomal subunit. Toxic proteins, such as *ldrD*, are toxic at high levels and can kill cells or inhibit growth, which functions to reduce the host cell’s viability [15].

### *Salmonella enterica* (*S. enterica*)

In *S*. *enterica*, the MgtC virulence factor is involved in adaptation to low magnesium environments. The hydrophobic peptide MgrR, binds to MgtC, causing its degradation by the FtsH protease [9].

### *Bacillus subtilis* (*B. subtilis*)

The 46 aa Sda micropeptide, expressed by *B*. *subtilis*, represses sporulation when replication initiation is impaired. By inhibiting the histidine Kinase KinA, Sda prevents the activation of the transcription factor Spo0A, which is required for sporulation [10].

### *Staphylococcus aureus* (*S. aureus*)

In *S*. *aureus*, there are a group of micropeptides, 20–22 aa, that are excreted during host infection to disrupt neutrophil membranes, causing cell lysis. These micropeptides allow the bacterium to avoid degradation by the human immune systems' main defenses [16,17].

## Eukaryotic examples

Micropeptides have been discovered in eukaryotic organisms from *Arabidopsis thaliana* to humans. They play diverse roles in tissue and organ development, as well as maintenance and function once fully developed. While many are yet to be functionally characterized, and likely more remain to be discovered, below is a summary of recently identified eukaryotic micropeptide functions.

### *Arabidopsis thaliana* (*A. thaliana*)

The *POLARIS (PLS)* gene encodes a 36 aa micropeptide. It is necessary for proper vascular leaf patterning and cell expansion in the root. This micropeptide interacts with developmental PIN proteins to form a critical network for hormonal crosstalk between auxin, ethylene, and cytokinin [18,19,20].

*ROTUNDIFOLIA (ROT4*) in *A*. *thaliana* encodes a 53 aa peptide, which localizes to the plasma membrane of leaf cells. The mechanism of ROT4 function is not well understood, but mutants have short rounded leaves, indicating that this peptide may be important in leaf morphogenesis [21].

### *Zea mays* (*Z. mays*)

Brick1 (Brk1) encodes a 76 aa micropeptide, which is highly conserved in both plants and animals. In *Z*. *mays*, it was found to be involved in morphogenesis of leaf epithelia, by promoting multiple actin-dependent cell polarization events in the developing leaf epidermis [22]. Zm401p10 is an 89 aa micropeptide, which plays a role in normal pollen development in the tapetum. After mitosis it also is essential in the degradation of the tapetum [23]. Zm908p11 is a micropeptide 97 aa in length, encoded by the *Zm908* gene that is expressed in mature pollen grains. It localizes to the cytoplasm of pollen tubes, where it aids in their growth and development [24].

### *Drosophila melanogaster* (*D. melanogaster*)

The evolutionarily conserved polished rice (*pri*) gene, known as *tarsal-less (tal) in D*. *melanogaster*, is involved in epidermal differentiation. This polycistronic transcript encodes four similar peptides, which range between 11–32 aa in length. They function to truncate the transcription factor Shavenbaby (Svb). This converts Svb into an activator that directly regulates the expression of target effectors, including *miniature (m)* and *shavenoid (sha)*, which are together responsible for trichome formation [25].

### *Danio rerio* (*D. rerio*)

The *Toddler* (*tdl)* gene is believed to be important for embryogenesis, and is specifically expressed during late blastula and gastrula stages. During gastrulation, it is critical in promoting the internalization and animal-pole directed movement of mesendodermal cells. After gastrulation, it is expressed in the lateral mesoderm, endoderm, as well as the anterior, and posterior, notochord. Although it is annotated as a lncRNA in zebrafish, mouse, and human, the 58 aa sORF was found to be highly conserved in vertebrates [26,27].

### *Mus musculus* (*M. musculus*)

Myoregulin (Mln) is encoded by a gene originally annotated as a lncRNA. Mln is expressed in all 3 types of skeletal muscle, and works similarly to the micropeptides phospholamban (Pln) in the cardiac muscle and sarcolipin (Sln) in slow (Type I) skeletal muscle. These micropeptides interact with sarcoplasmic reticulum Ca^2+^-ATPase (SERCA), a membrane pump responsible for regulating Ca^2+^ uptake into the sarcoplasmic reticulum (SR). By inhibiting Ca^2+^ uptake into the SR, they cause muscle relaxation. Similarly, the endoregulin (ELN) and another-regulin (ALN) genes code for transmembrane micropeptides that contain the SERCA binding motif, and are conserved in mammals [7].

Myomixer (Mymx) is encoded by the gene *Gm7325*, a muscle-specific peptide, 84 aa in length, which plays a role during embryogenesis in fusion and skeletal muscle formation. It localizes to the plasma membrane, associating with a fusogenic membrane protein, Myomaker (Mymk). In humans, the gene encoding Mymx is annotated as uncharacterized *LOC101929726*. Orthologs are found in the turtle, frog and fish genomes as well [8].

### *Homo sapiens* (*H. sapiens*)

In humans, NoBody (non-annotated P-body dissociating polypeptide), a 68 aa micropeptide, was discovered in the long intervening noncoding RNA (lincRNA) *LINC01420*. It has high sequence conservation among mammals, and localizes to P-bodies. It enriches proteins associated with 5’ mRNA decapping. It is thought to interact directly with Enhancer of mRNA Decapping 4 (EDC4) [28].

The *C7orf49* gene, conserved in mammals, when alternatively spliced is predicted to produce three micropeptides. MRI-1 was previously found to be a modulator of retrovirus infection. The second predicted micropeptide, MRI-2, may be important in non-homologous end joining (NHEJ) of DNA double strand breaks. In Co-Immunoprecipitation experiments, MRI-2 bound to Ku70 and Ku80, two subunits of Ku, which play a major role in the NHEJ pathway [29].

The 24 amino acid micropeptide, Humanin (HN), interacts with the apoptosis-inducing protein Bax (Bcl2-associated X protein). In its active state, Bax undergoes a conformational change which exposes membrane-targeting domains. This causes it to move from the cytosol to the mitochondrial membrane, where it inserts and releases apoptogenic proteins such as cytochrome c. By interacting with Bax, HN prevents Bax targeting of the mitochondria, thereby blocking apoptosis [30].

A micropeptide of 90aa, ‘Small Regulatory Polypeptide of Amino Acid Response’ or SPAAR, was found to be encoded in the lncRNA *LINC00961*. It is conserved between human and mouse, and localizes to the late endosome/lysosome. SPAR interacts with four subunits of the v-ATPase complex, inhibiting mTORC1 translocation to the lysosomal surface where it is activated. Down-regulation of this micropeptide enables mTORC1 activation by amino acid stimulation, promoting muscle regeneration [31].
